# Lactic Acid Bacterial Supplementation Ameliorated the Lipopolysaccharide-Induced Gut Inflammation and Dysbiosis in Mice

**DOI:** 10.3389/fmicb.2022.930928

**Published:** 2022-06-13

**Authors:** Ruchika Bhatia, Shikha Sharma, Sanjay Kumar Bhadada, Mahendra Bishnoi, Kanthi Kiran Kondepudi

**Affiliations:** ^1^Healthy Gut Research Group, Centre for Excellence in Functional Foods, Food and Nutrition Biotechnology Division, National Agri-Food Biotechnology Institute, Sahibzada Ajit Singh Nagar, India; ^2^Department of Biotechnology, Panjab University, Chandigarh, India; ^3^Department of Endocrinology, Post Graduate Institute of Medical Education and Research (PGIMER), Chandigarh, India; ^4^Regional Centre of Biotechnology, Faridabad, India

**Keywords:** lipopolysaccharide, inflammation, *Lactobacillus*, intestinal permeability, tight junction proteins

## Abstract

Lipopolysaccharide (LPS), a gut-transmitted endotoxin from Gram-negative bacteria, causes inflammatory diseases leading to the loss of gut barrier integrity and has been identified as a major pathogenic stimulator in many dysfunctions. Hence, supplementation with probiotics is believed to be one of the most effective strategies for treating many inflammatory gut disorders. Although probiotics are known to have a variety of therapeutic characteristics and to play a beneficial role in host defense responses, the molecular mechanisms by which they achieve these beneficial effects are unknown due to species- and strain-specific behaviors. Therefore, in this study, the protective role of five indigenous lactic acid bacterial strains in ameliorating LPS-induced gut barrier impairment in the C57BL/6 mice model was elucidated. *Lacticaseibacillus rhamnosus* LAB3, *Levilactobacillus brevis* LAB20, and *Lactiplantibacillus plantarum* LAB31 were isolated from infant feces; *Pediococcus acidilactici* LAB8 from fermented food (Bekang); and *Lactiplantibacillus plantarum* LAB39 from beetroot. Intraperitoneal injection of LPS (10 mg/kg of body weight) increased the levels of lipocalin and serum markers TNF-α, IL-6, and IL-1β, and the overall disease activity index in the treated group. Furthermore, gene expression of *NF-kB*, *IL-12*, and *Cox-2*; mucin-producing genes *Muc-2* and *Muc-4*; and intestinal alkaline phosphatase (IAP) was deleteriously altered in the ileum of LPS-treated mice. Furthermore, LPS also induced dysbiosis in gut microbiota where higher abundances of *Klebsiella*, *Enterobacter*, and *Salmonella* and decreased abundances of *Lactobacillus*, *Bifidobacteria*, *Roseburia*, and *Akkermansia* were observed. Western blotting results also suggested that LPS treatment causes the loss of gut barrier integrity relative to the pre-supplementation with LAB strains, which enhanced the expression of tight junction proteins and ameliorated the LPS-induced changes and inflammation. Taken together, the study suggested that LAB3 and LAB39 were more potent in ameliorating LPS-induced gut inflammation and dysbiosis.

## Graphical Abstract

Lactic acid bacteria elevates the IAP expression, intestinal gut integrity and prevents the translocation of LPS into the systemic circulation, and reduces gut inflammation. 
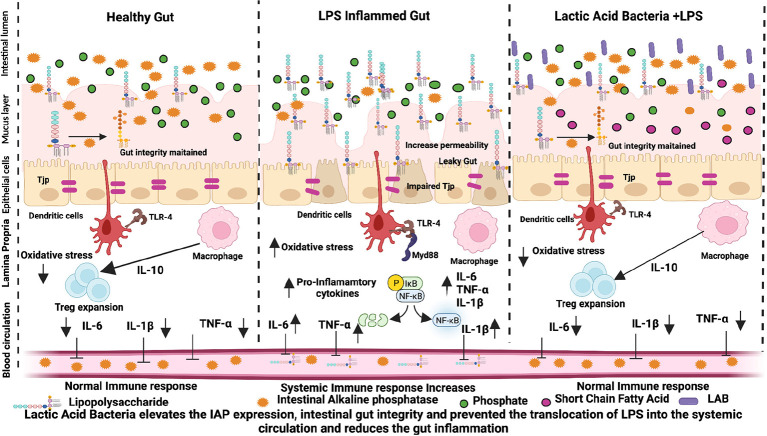

## Introduction

The gastrointestinal tract is populated by a diverse variety of microorganisms, such as bacteria, viruses, fungi, and other single-celled creatures, all of them together known as gut microbiota (Monteros et al., 2021). A harmonious association between the host and the microbiota is critical for intestinal homeostasis, absorption of nutrients, and synthesis of important biomolecules through a wide array of enzymes with biosynthetic abilities. Hence, imbalance in the microbial community composition (dysbiosis) contributes to the overgrowth of gut pathogens, which destabilizes the whole intestinal mucosa and triggers a cascade of strong inflammatory responses (Petrella et al., 2021). Moreover, the acute inflammatory response is vital for tissue regeneration, infection, trauma, noxious stimuli, and elimination of injurious agents from the damaged tissue (Wang et al., 2020). Among the various known factors, microorganisms and their cellular components are considered to be the foremost common cause that triggers inflammation. LPS is an important glycoprotein derived from the outer cell wall membrane of Gram-negative bacteria consisting of hydrophobic (lipid A) and hydrophilic (polysaccharide O-antigen and carbohydrate core) components, which elicit a pro-inflammatory response, making the intestinal wall highly relaxed and permeable causing a “leaky gut” which further allows the translocation of bacteria and their metabolites, such as toxins, to other extra-intestinal organs (Kostelac et al., 2021). LPS binds to the toll-like receptor (TLR-4), resulting in the activation of mitogen-activated protein kinases (MAPKs) and nuclear factor kappa B (NF-κB) pathways (Kawano et al., 2016).

Mucin and the epithelial layer constitute intestinal alkaline phosphatase (IAP) and antibacterial proteins, which together represent the functional barrier of the immune system (An et al., 2021). IAP and glycoproteins are attached to the plasma membrane and hydrolyze multiple monophosphates at alkaline pH with the release of inorganic phosphates. IAP dephosphorylates substrates, such as lipopolysaccharide (LPS) and other pathogen-associated molecular patterns, and plays a pivotal role in reducing local intestinal inflammation and maintaining gut barrier integrity (Singh et al., 2020; Santos et al., 2022). Moreover, IAP has lately gained prominence in the prevention and treatment of many disorders associated with leaky gut. Therefore, the utilization of IAP as a potential biomarker in the amelioration of many inflammatory, alcoholic, and non-alcoholic fatty liver diseases by inhibiting TLR4/LPS signaling is an active area of research (Son et al., 2018).

Although many therapeutic agents which can help in reducing inflammatory conditions are available, they are often associated with many side effects. Therefore, supplementation with probiotics has emerged as an important dietary intervention strategy in reinforcing the intestinal gut integrity for treating many gut-associated complications. In a recent study, immunomodulatory effects of *Lactobacillus reuteri* ZJ617 and ZJ615, with high and low adhesive abilities, and *Lactobacillus rhamnosus* GG (LGG) in mice stimulated with lipopolysaccharide (LPS) have been evaluated (Gao et al., 2016; Chaiyasut et al., 2017). Total surface proteins derived from *Lpb. plantarum* MTCC 5690, *Lm. fermentum* MTCC 5689, and *L. acidophilus* NCFM have been reported for their ameliorative role in TNNBS colitis and DSS mouse models (Chandhni et al., 2021). Owing to the strain-dependent differences in the probiotic potentials, it is imperative to test the strains for their ability to prevent inflammation in inflammatory disease models (Cristofori et al., 2021). Many cell line and rodent studies highlighted the protective role of lactic acid bacteria in mitigating gastrointestinal disorders (Oh et al., 2018; Zhao et al., 2019; Cristofori et al., 2021), but on the other hand, studies on the anti-inflammatory potential of Indian-origin lactic acid bacterial strains are scarce as the majority of the studies revolves around the safety and toxicity of the indigenous probiotics (Pradhan et al., 2019). In this context, we have selected five indigenous LAB strains *Lacticaseibacillus rhamnosus* LAB3, *Pediococcus acidilactici* LAB8, *Levilactobacillus brevis* LAB20, *Lactiplantibacillus plantarum* LAB31, and *Lactiplantibacillus plantarum* LAB39 with an ability to inhibit inflammatory mediators through the suppression of MAPK and NF-kB signaling pathways in the murine macrophages. Furthermore, these strains also prevented TNF-α and LPS-induced IL-8 production by human intestinal epithelial cells and possess probiotic features (unpublished data). After evaluating their potential anti-inflammatory role *in vitro*, the next step was to study their effect in the experimental mice model. Hence, in this study, prophylactic potential of the five indigenous anti-inflammatory lactic acid bacteria in curtailing LPS-induced inflammation in the C57BL/6 mice was evaluated.

## Materials and Methods

### Materials

Lipopolysaccharide (*E. coli* 055: B5), SDS, EDTA, hydroxylamine hydrochloride, thiobarbituric acid, RNA later, Bradford reagent, and protease inhibitor cocktail were procured from Sigma-Aldrich (St. Louis, MO, United States). TRIzol was purchased from Invitrogen, California, United States. de Man, Rogosa, and Sharpe (MRS) agar, glass slides, paraffin, hematoxylin, eosin, alcian blue, glycerol, hydrogen peroxide (H_2_O_2_), sodium bicarbonate (NaHCO_3_), sodium carbonate (Na_2_CO_3_), sulfosalicylic acid, formalin, potassium chloride (KCl), and other chemicals were from HiMedia Laboratories, Mumbai, India; mouse-specific lipocalin-2, IL-6, IL-1β, and TNF-α ELISA kits were procured from Ray Biotech, Norcross, United States. SYBR-Green master mix and cDNA synthesis kits were purchased from Bio-Rad, United States. HPLC with a diode array detector was from Agilent Technologies, Singapore. NucleoSpin DNA Stool kits were procured from Macherey-Nagel, Düren, Germany. Antibodies (occludin, zona occludin, claudin, GAPDH, Muc-4, and Alexa Fluor 488) from Cell Signaling Technology (Danvers, MA, United States). HRP (horseradish peroxidase)-conjugated secondary antibody and DNase treatment AM1906 were purchased from Thermo Fisher, Leica CTR6, and Leica Biosystems, Germany. A confocal microscope was purchased from Carl Zeiss LSM-880, Germany.

### Methodology

#### Bacterial Strains and Culture

LAB strains *Lacticaseibacillus rhamnosus* LAB3, *Levilactobacillus brevis* LAB20, and *Lactiplantibacillus plantarum* LAB31 isolated previously from infant feces, *Pediococcus acidilactici* LAB8 from fermented food (bekang), and *Lactiplantibacillus plantarum* LAB39 from beetroot were used in the present study. Trypticase soy broth (TSB) containing 15% sterile glycerol was used to activate the bacterial with strains on MRS plates and incubated under aerobic conditions for 48 h at 37°C. A single colony of each strain was sub-cultured consecutively three times in MRS broth at 37°C and incubated for 24 h. After the third transfer, the cultures were cold-centrifuged at 6,000×g, and the pellets thus attained were washed and resuspended in sterile PBS, obtained as stock culture in TSB consisting of 15% glycerol, and stored at –80°C. At the time of dosing, the cells were centrifuged and suspended in 200 μl of PBS to get 1 × 10^10^ CFU/mouse and were administered by gavage to each mouse every day from the start to the end of the experiment.

#### Prevention of Lipopolysaccharide-Induced Inflammation in C57BL6/J Mice

**FIGURE 1:**
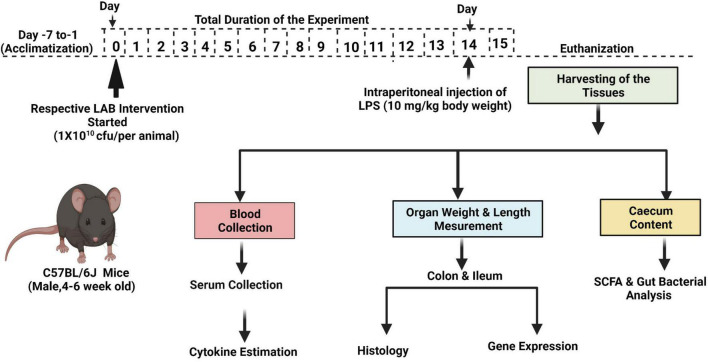
Scheme of the study.

The animal study was approved by the Institutional Animal Ethics Committee (IAEC), National Agri-Food Biotechnology Institute (NABI), Mohali, Punjab (NABI/2039/CPCSEA/IAEC/2020/11). The experiments were conducted as per the Committee for the Purpose of Control and Supervision on Experiments on Animals (CPCSEA). A brief protocol of the experiment is given in Figure 1. Male C57BL6/J black mice (aged 4–6 weeks) were procured from IMTech Centre for Animal Resources and Experimentation (iCARE), Chandigarh, India, and housed in the Animal Experimentation Facility, NABI. The animals were kept under standard experimental conditions, that is, 20 ± 2°C; maintained on a 12-h light/dark cycle; and acclimatized for 7 days. The mice were fed with a standard pellet diet and water during the entire experimental period.

##### Experimental Plan

Age- and weight-matched mice were randomized into 12 experimental groups, with five mice in each group. The experimental groups were designated as control and LPS; LAB3, LAB8, LAB20, LAB31, and LAB39 *per se* groups; and the prevention groups LAB3 + LPS, LAB8 + LPS, LAB20 + LPS, LAB31 + LPS, and LAB 39 + LPS, respectively. Each mouse in the *per se* and prevention groups was fed with respective strains orally by gavage on every day until the 15th day. In the control and LPS groups, the mice were administered with PBS as a vehicle. Inflammation was induced by a single intraperitoneal (i.p.) injection of LPS (10 mg/kg of body weight) to the LPS and prevention groups on the 14th day (Gao et al., 2016). After 24 h, that is, on the 15th day, blood was collected by cardiac puncture and allowed to clot at 4°C for 2 h and was centrifuged at 2,000×g for 10 min at 4°C to obtain serum samples, which were stored at –80°C until further use. Furthermore, after 24 h of LPS injection, the number of lactobacilli released in the feces and sticking to the ileal mucosa was checked in different growth media (*n* = 3) (Supplementary Figure 1). Induction of inflammation was ascertained by determining the level of lipocalin-2 in the feces using an ELISA kit following the instructions.

The overall disease activity index (DAI) was calculated using weight loss data (0, no loss; 2, ≤5% loss; 3, and ≤10% loss), stool consistency (0, normal; 2, loose; and 4, diarrhea), and gross bleeding (0, normal color; 2, reddish color; and 4, bloody stool) divided by three for each mouse in every group (Toump et al., 2014).

### Biochemical Analysis

#### Proximal Colon Tissue Homogenate Preparation

Snap-frozen proximal colon tissue (50 mg) was rinsed thoroughly with ice-cold PBS. A 10% (w/v) colon homogenate containing protease inhibitor cocktail was prepared in 0.1 M PBS (pH 7.4) and centrifuged at 8,000 × g at 4°C for 20 min. The supernatant obtained was collected and stored at −20°C. Protein concentrations were determined in the homogenates by using the Bradford method. Malondialdehyde, catalase, superoxide dismutase, and reduced glutathione were assayed, as described elsewhere (Kono, 1978; Singh et al., 2018).

#### Inflammatory Marker Analysis in the Serum

TNF-α, IL-6, IL-1β, IL-10, interferon (IFN-ɣ), C-reactive protein (CRP), and IL-22 levels were measured in serum using ELISA kits as per the instructions.

### Histopathological Analysis

Colon and ileum tissues were fixed in 10% phosphate-buffered formalin, embedded in paraffin, and sectioned at a 5 μm thickness using a microtome. The slides were processed with hematoxylin/eosin staining to visualize the tissue architecture and inflammatory cell infiltration. Alcian blue staining was performed on discrete sections to count the number of goblet cells. The stained sections were examined and photographed under a light microscope at 20X objective. At least eight sections, each of the colon and ileum, from three different animals per group were analyzed for histologic scoring. ImageJ software (NIH) was used for the analysis.

### RNA Extraction and Real-Time Quantitative PCR

RNA was extracted from the snap-frozen colon and ileum tissues using the TRIzol method (Singh et al., 2016). Approximately 50–60 mg of tissue samples were homogenized and kept at room temperature for 5 min, followed by 4°C centrifugation at 18,000×g for 10 min. Chloroform was added and centrifuged, and the supernatant thus obtained was pipetted out into a fresh Eppendorf tube, followed by the addition of isopropanol. The mixture was then centrifuged at 18,000×g for 20 min at 4°C to pellet down the RNA (as pellet) at the bottom of the Eppendorf tube. Then, 500 μl of 70% chilled ethanol was added to precipitate the RNA and centrifuged at 18,000×g for 10 min. The pellet was air-dried, and the precipitated RNA was suspended in nuclease-free water. The integrity of the RNA was evaluated using agarose (1.2%) gel electrophoresis, and DNase treatment was carried out to remove the genomic DNA contamination and then was quantified on Nanodrop. Pure and intact total RNA (1 μg) were reverse-transcribed to complementary DNA using a single-stranded cDNA synthesis kit as per the manufacturer’s instructions. Relative gene expression of various genes was determined using an iTaq Universal SYBR Green Supermix. RT-PCR was performed under the following conditions: 95°C for 2 min for initial denaturation, 95°C for 5 s and annealing/extension at 60°C for 30-s × 40 cycles, final extension at 60°C for 5 min, and melt curve analysis between 60 and 95°C with 0.5°C/5-s increment. β-Actin was used as a housekeeping gene; the C_T_ values for all wells were exported into an Excel spreadsheet, and data were analyzed using the 2^–ΔΔCt^ method. The list of primers used in the experiment is given in Supplementary Table 1.

### Relative Abundance of Selected Gut Bacteria in Cecal Contents

Genomic DNA was extracted from approximately 100 mg of cecal contents of each mouse using the NucleoSpin DNA Stool kit as per the manufacturer’s instructions. The purified DNA was eluted, and the Infinite M200 PRO NanoQuant was used for DNA quantification. Relative abundance of bacteria was determined using a Universal SYBR Green Supermix under the following RT-PCR conditions: 95°C for 2 min, 40 cycles of 95°C for 5 s, and 60°C for 30 s performed using appropriate bacterial primers (Supplementary Table 2). For normalization, total bacterial primer was used, and the results were expressed as relative fold change of bacterial DNA abundance compared with the control group using the 2^–ΔΔCt^ method.

### Analysis of Short-Chain Fatty Acid in the Cecal Content

Approximately 50 mg of cecal content (stored at −80°C) was extracted with 500 μl of acidified water (adjusted to pH 2), followed by vigorous vortexing and incubation for 10 min at room temperature. The resultant mixture was centrifuged at 3,850×g for 20 min at 4°C and filtered through 0.2-μm nylon membrane filters. The Agilent HPLC system assembled with a controller pump, autosampler unit, and diode array detector set at 210 nm was used for SCFA estimation. Separation of lactate, acetate, propionate, and butyrate was attained using an anion exchange column, along with a guard column made of monodisperse, sulfonated styrene–divinylbenzene copolymer at 60°C. A mixture of 0.1% formic acid prepared in Milli-Q water was used as a mobile phase. The injection volume was 20 μl, and five extracts from each group were injected. Data thus obtained were processed with EZChrom Elite software. Using various known concentrations of the peak, areas of propionate, acetate, butyrate, and lactate were calculated, and a calibration curve was drawn for the same. Data were represented as micromolar concentration of SCFAs per milligram of the cecal content.

### Immunohistochemistry

Formalin-fixed colon sections were cleaned with PBS, deparaffinized, and subjected to a series of rehydration steps. The tissue sections were processed for antigen retrieval (citrate buffer pH 6.0) at 90°C for 30 min, followed by blocking (5% goat serum in PBST, Tween-20, 0.1% w/v). The Muc-4 Primary antibody (1: 200X dilution in 2.5% goat serum) was added and incubated in a humidified chamber at 4°C overnight. After washing three times in PBS-T for 5 min, Alexa Fluor 488-coupled secondary antibody (1:2,000X dilution) was added and incubated at room temperature for 2 h. PureBlu DAPI (1 μg/ml) was used to counterstain the cell nuclei. Imaging was performed using a confocal microscope, and the images obtained were analyzed using ImageJ software. The expression of the Muc-4 antibody was normalized to DAPI, and the mean values were expressed as mean ± SEM, with *n* = 3/group.

### Western Blotting

Approximately 50 mg of colon tissues were homogenized in RIPA buffer having a protease inhibitor cocktail. The lysate was centrifuged at 8,000×g for 30 min, and the supernatant obtained was used in the blotting experiment. The bicinchoninic acid (BCA) assay method was used to determine the protein content in the homogenates. Approximately 15 μg of protein was separated on 10% SDS-PAGE, along with a pre-stained protein marker for reference. After electrophoresis, the gel was transferred onto the PVDF membrane in an ice-cold buffer containing Tris-buffered saline (10 mM Tris-Cl, pH 7.4) containing 0.5% Tween 20 (TBST). After that, the membrane was treated with 5% non-fat dry milk at room temperature for 2 h to avoid non-specific binding. Later, the membrane was washed three times with TBST; probed with primary antibodies at 1:1,000 dilution for occludin, zona occludin, claudin, and β-actin, respectively; and incubated at 4°C overnight. After rinsing with TBST, the immunoblots were incubated with horseradish peroxidase-conjugated anti-rabbit immunoglobulin G secondary antibody at 1:3,000 dilution at room temperature for 2 h. After subsequent washing with TBST, blots were detected using the enhanced chemiluminescence (ECL) detection system. The relative densities of each protein band were determined using ImageJ software. The expressions of occludin, claudin, and zona occludin were normalized to β-actin. The mean values were expressed as mean ± SEM, with *n* = 5/group.

### Statistical Analysis

GraphPad Prism 8 software was used, and all the values were represented as mean ± SEM. The statistical significance between the different experimental groups was analyzed using a one-way analysis of variance (ANOVA), followed by Tukey’s *post hoc* test. *p* ≤ 0.05 was accounted as significant.

## Results

### *Lactobacillus* Supplementation Prevented the Lipopolysaccharide-Induced Reduction in Body Weight, Colon Length, Colon Weight, and Spleen Weight

**FIGURE 2:**
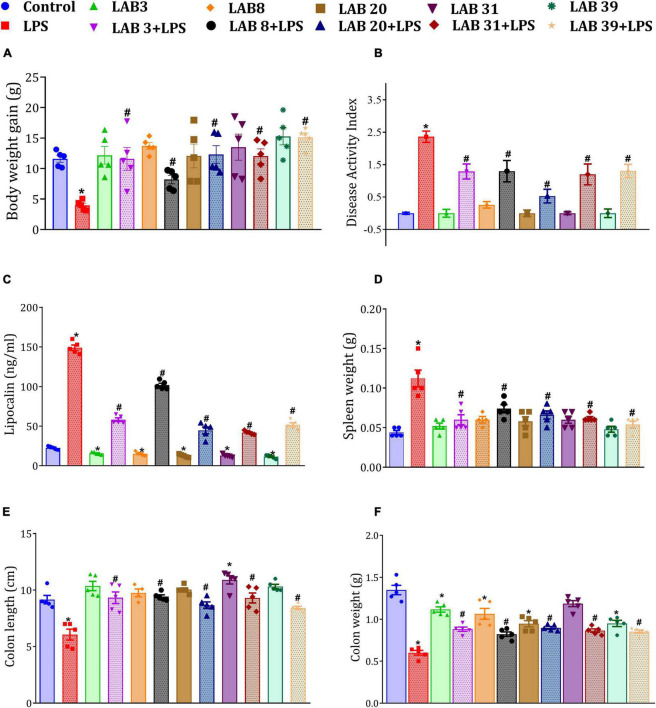
Effect of LAB supplementation on: **(A)** Weight gain; **(B)** disease activity index; **(C)** fecal lipocalin levels; **(D)** spleen weight; **(E)** colon length; and **(F)** colon weight. Data were analyzed by one-way ANOVA with Tukey’s *post hoc* test (*P* ≤ 0.05). *Significant relative to control; #significant relative to LPS-treated animals (*N* = 5).

Lipopolysaccharide treatment showed loss in body weight (Figure 2A), promoted the disease activity index (Figure 2B), increased fecal lipocalin levels (Figure 2C), increased the weight of the spleen (Figure 2D), and decreased the colonic length (Figure 2E) and colonic weight (Figure 2F) relative to the control mice. The mice pre-supplemented with LAB3, LAB20, LAB31, and LAB39 before the LPS treatment showed no decrease in body weight relative to the LPS-treated mice (Supplementary Figure 2A). It was also observed that LAB *per se*-supplemented groups showed normal spleen weight and normal colonic length and colonic weight compared to the normal mice (Figures 2D–F). The representative colon length images are included in Supplementary Figure 2B.

### *Lactobacillus* Supplementation Prevented the Increase in Oxidative Stress and Systemic Inflammation Caused by Lipopolysaccharide

**FIGURE 3:**
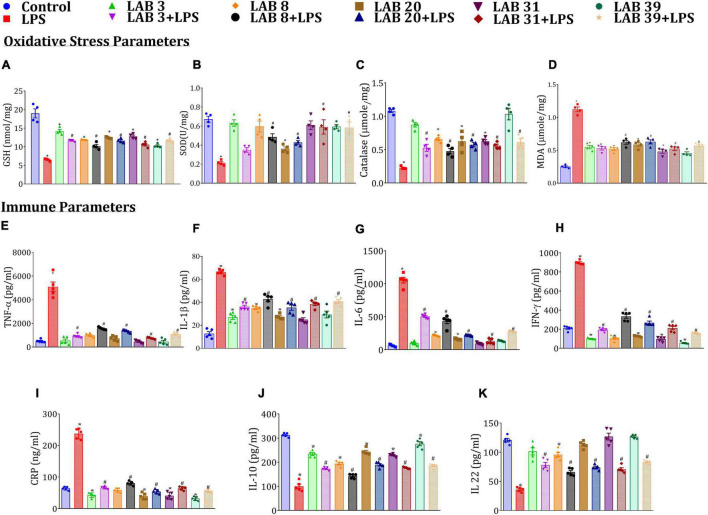
Effect of LAB supplementation on the oxidative stress and systemic inflammation caused by LPS: **(A)** GSH; **(B)** SOD; **(C)** catalase; **(D)** MDA; **(E)** TNF-α; **(F)** IL-1β; **(G)** IL-6; **(H)** IFN-γ; **(I)** CRP; **(J)** IL-10; and **(K)** IL-22. Data were analyzed by one-way ANOVA with Tukey’s *post hoc* test (*P* ≤ 0.05). *Significant relative to control;^ #^significant relative to LPS-treated animals (*N* = 4 for oxidative stress parameters; *N* = 5 for immune markers).

Lipopolysaccharide treatment decreased the SOD, GSH, and catalase levels but increased MDA, systemic immune levels of TNF-α, IL-1β, IL-6, IFN-ɣ, and CRP relative to the normal mice (Figures 3A–I). The mice pre-supplemented with LAB strains prior to LPS treatment showed elevated levels of SOD, GSH, and catalase and decreased levels of the immune markers relative to the LPS-treated mice. Furthermore, the mice supplemented with LAB *per se* had lower levels of these cytokines relative to normal mice. Interestingly, the levels of IL-22 and IL-10 were higher in LAB3-, LAB8-, LAB20-, LAB31-, and LAB39-pre-supplemented groups than in the LPS-treated group (Figures 3J,K), while their production in the LAB *per se*-supplemented group remains same as that of the normal mice.

### *Lactobacillus* Supplementation Prevented the Lipopolysaccharide-Induced Ileal and Colonic Tissue Damage

**FIGURE 4:**
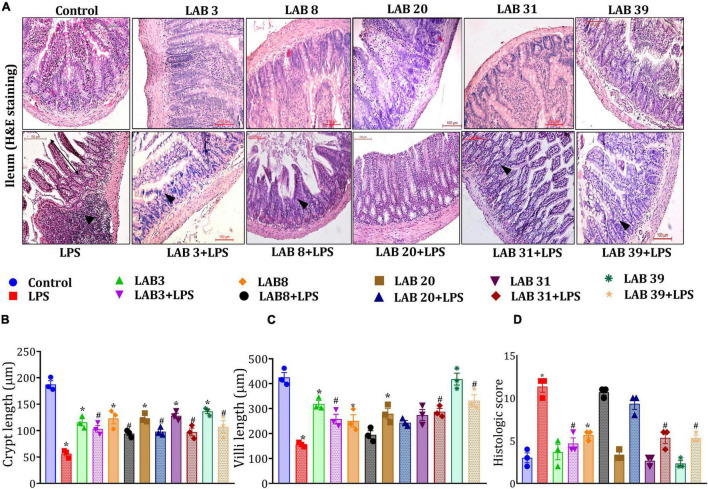
Effect of LAB supplementation on the ileum architecture: **(A)** Hematoxylin–eosin staining images; **(B)** crypt length; **(C)** villi length; and **(D)** histologic score. Data were analyzed using one-way ANOVA with Tukey’s *post hoc* test (*p* ≤ 0.05). *Significant relative to control; #significant relative to the LPS group (*N* = 3); (arrows indicate the neutrophil infiltration; *r*: 200 μm, 200X).

Histologic examinations of the tissue sections after hematoxylin and eosin staining suggested (Figure 4A) that LPS treatment altered the normal morphology of the ileum, that is, reduced the length of the villus and crypts, showed a higher number of neutrophil infiltration, and overall increased the histologic score compared to the normal mice (Figures 4B–D). In addition, pre-supplementation with LAB3 and LAB39 prior to the LPS treatment profoundly prevented these changes in comparison to LAB8 and LAB20 groups.

**FIGURE 5:**
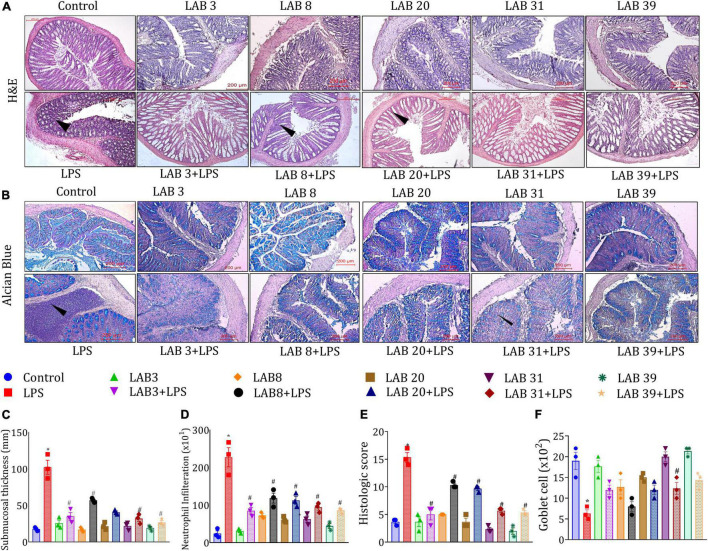
Effect of LAB supplementation on the colon architecture: **(A)** Hematoxylin–eosin; **(B)** alcian blue staining images; **(C)** submucosa thickness; **(D)** neutrophil infiltration; **(E)** colon histologic score; and **(F)** goblet cell. Data were analyzed using one-way ANOVA with Tukey’s *post hoc* test (*p* ≤ 0.05). *Significant relative to control; #significant relative to the LPS group (*N* = 3); (arrows indicate the neutrophil infiltration; *r*: 200 μm, 200X).

The histologic examination based on H&E staining showed that LPS treatment disturbed the colonic integrity (Figure 5A), decreased the number of goblet cells (Figure 5B), increased submucosa thickness (Figure 5C), increased the number of infiltrated neutrophils in the epithelial layer (Figure 5D), and overall increased the histologic score (Figure 5E) compared to the normal mice. Furthermore, pre-supplementation with LAB strains prior to the LPS treatment also increased the count of goblet cells, as depicted in Figure 5F. Thus, the overall finding suggested that LAB pre-supplementation prevented the harmful changes in the LPS-treated group.

### *Lactobacillus* Supplementation Beneficially Modulated the Gene Expression in the Colon and Ileum Tissues

**FIGURE 6:**
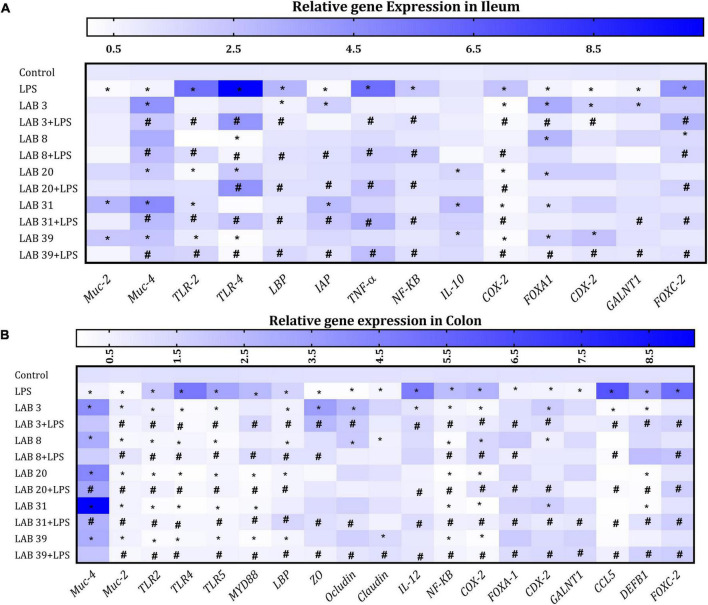
Effect of LAB supplementation on the relative gene expression in the ileum and colon: Heat map overview of genes in **(A)** ileum and **(B)** colon. Data were analyzed using one-way ANOVA with Tukey’s *post hoc* test (*p* ≤ 0.05). *Significant relative to control; #significant relative to the LPS group (*N* = 4).

Lipopolysaccharide treatment increased the expression of *TLR-4*, *TLR2*, *TNF-*α, *NF-kB*, and *Cox2* but decreased the expression of mucus-producing genes *MUC-4* and *MUC-2* and goblet markers *CDX2* and *FOXA1* in the ileum compared to the normal mice. Furthermore, LPS treatment increased the expression of *FOXC-2* and *LBP* and decreased the expression of *IAP* and *IL-10* relative to the normal mice. Increased expression of *IAP* and decreased expression of *LBP* and *FOXC-2* were observed in LAB-pre-supplemented groups compared to the LPS group. The expression of goblet markers *CDX2* and *FOXA1* was high in LAB3, LAB8, and LAB20 *per se*-supplemented groups relative to the control mice. The levels of pro-inflammatory cytokines and *TLR-2* and *TLR-4* were decreased in LAB3 + LPS-, LAB31 + LPS-, and LAB39 + LPS-pre-supplemented groups relative to the LPS group (Figure 6A).

In the colon, LPS treatment elevated the expression of *TLR4*, *TLR2*, and *TLR5* but decreased the expression of mucus-producing genes *MUC-4* and *MUC-2* and goblet cell markers *FOXA1* and *CDX2* compared to the control. The expression of tight junction proteins *ZO*, *occludin*, and *claudin* was decreased in LPS-treated groups compared to the LAB3 + LPS- and LAB39 + LPS-pre-supplemented groups. The expression of *FOXC-2*, *MYD88 CCL5*, *DEFB1*, *LBP*, *IL-12*, *NF-kB*, and *COX-2* genes was increased in the LPS group compared to the LAB-pre-supplemented groups (Figure 6B).

### *Lactobacillus* Supplementation Beneficially Modulated the Selected Gut Bacterial Abundances

**FIGURE 7:**
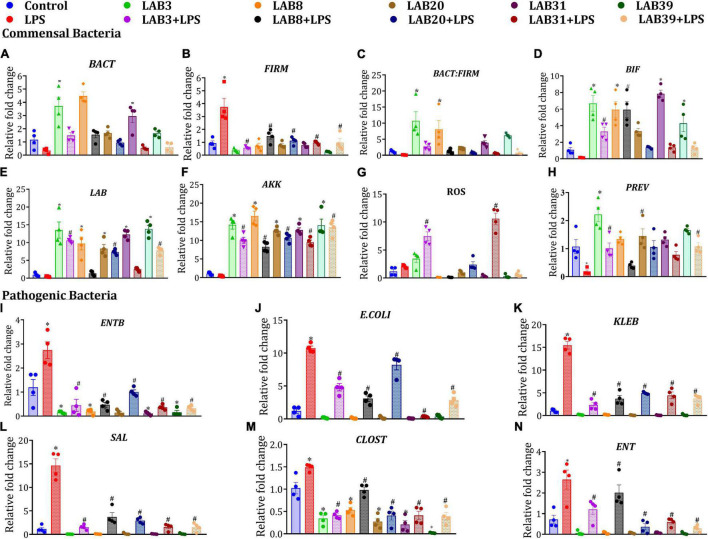
Effect of LAB supplementation on the selected gut bacterial abundances: **(A)** *BACT*; **(B)** *FIRM*; **(C)** *BACT: FIRM*; **(D)** *BIF*; **(E)** *LAB*; **(F)** *AKK*; **(G)** *ROS*; **(H)** *PREV*; **(I)** *ENTB*; **(J)** *E. coli*; **(K)** *KLEB*; **(L)** *SAL*; **(M)** *CLOST*; and **(N)** *ENT*. Data were analyzed by one-way ANOVA with Tukey’s *post hoc* test (*P* ≤ 0.05) and are represented as mean ± SEM (*N* = 4). *LAB*, *Lactobacillus*; *BIF*, *Bifidobacteria*; *ROS*, *Roseburia*; *AKK*, *Akkermansia*; *FIRM*, *Firmicutes*; *BACT*, *Bacteroidetes*; *PREV*, *Prevotella*; *E. coli*; *KLEB*, *Klebsiella*; *SAL*, Salmonella; *ENTB*, *Enterobacteriaceae*; *CLOST*, *Clostridium*; *ENT*, *Enterobacter.* *Significant relative to control; # significant relative to LPS group (*N* = 4).

Lipopolysaccharide treatment decreased the abundance of selected beneficial gut bacteria, such as *Lactobacillus*, *Bifidobacteria*, *Roseburia*, *Akkermansia*, Bacteroidetes, and *Prevotella*, and increased the abundance of pathogenic bacteria, such as *E. coli*, *Klebsiella*, *Salmonella*, *Clostridium*, and Firmicutes, relative to the normal mice. Furthermore, pre-supplementation in LAB3 + LPS, LAB8 + LPS, LAB 31 + LPS groups enhanced the abundance of *Bacteroidetes*, *Lactobacillus*, *Bifidobacterium*, and *Akkermansia* but declined the abundance of *Firmicutes* relative to LPS-treated mice (Figures 7A–H). On the other hand, in the *per se* LAB-supplemented groups, decreased abundance of *Enterobacter* and *Clostridium* was observed relative to the control group (Figures 7I–N).

### *Lactobacillus* Supplementation Improved the Short-Chain Fatty Acid Production in Lipopolysaccharide-Treated Mice

**FIGURE 8:**
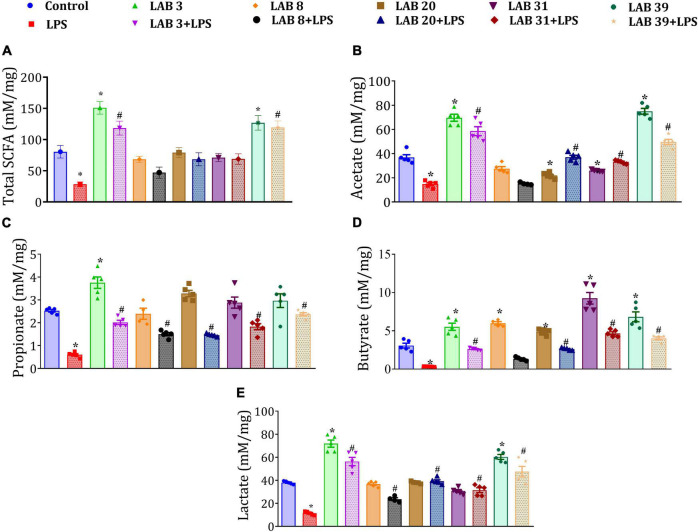
Effect of LAB supplementation on the short-chain fatty acid (SCFA) production: **(A)** Total SCFA production; **(B)** acetate; **(C)** propionate; **(D)** butyrate; and **(E)** lactate. Data were analyzed by one-way ANOVA with Tukey’s *post hoc* test (*P* ≤ 0.05) and are represented as mean ± SEM (*N* = 5). *significant relative to control; ^#^significant relative to the LPS group.

Lipopolysaccharide treatment decreased the levels of total SCFAs—acetate, propionate, butyrate, and lactate in the cecal content relative to the control mice. Furthermore, the mice pre-supplemented with LAB prior to LPS treatment showed improved levels of acetate, propionate, butyrate, and lactate relative to the LPS group. The mice supplemented with LAB *per se* showed increased levels of acetate, propionate, butyrate, and lactate relative to the control mice (Figures 8A–E).

Hierarchical clustering analysis was performed in all the LAB-pre-supplemented group before the LPS treatment using 25 evaluation parameters represented in rows and columns, respectively, assessed in the study. The heat map of unsupervised hierarchical clustering observed that among all the selected LAB-pre-supplemented groups, LAB3 + LPS and LAB39 + LPS are closely clustered in the same group and showed a lower trend in the inflammatory cytokine markers like IFN-ɣ, CRP, and IL-1β and increased levels of anti-inflammatory markers IL-10 and IL-22 relative to other groups. Furthermore, the total SCFA production was also significantly increased in the LAB3 and (Supplementary Figure 3), which are closely clustered, while no significant difference was observed in different LAB treatment groups, suggesting that all LAB strains confer protection against the LPS-induced inflammation and dysbiosis. Hence, on the basis of inflammatory markers and gut bacterial dysbiosis, we have shortlisted LAB3 and LAB39 to further study their mechanism in mitigating LPS inflammation.

### Modulation of Muc-4 Expression in Lipopolysaccharide-Induced Inflammation by *Lactobacillus* Supplementation

Immunohistochemical analysis of the mice in LPS-treated groups showed a decreased expression of Muc-4 in the colon compared to the normal mice. The mice pre-supplemented with LAB39 strains prior to the LPS treatment showed increased Muc-4 expression relative to the LPS-treated mice. The mice supplemented with LAB *per se* showed higher expression than the control mice (Supplementary Figures 4A,B).

### *Lactobacillus* Supplementation Enhanced the Tight Junction Proteins Expression

**FIGURE 9:**
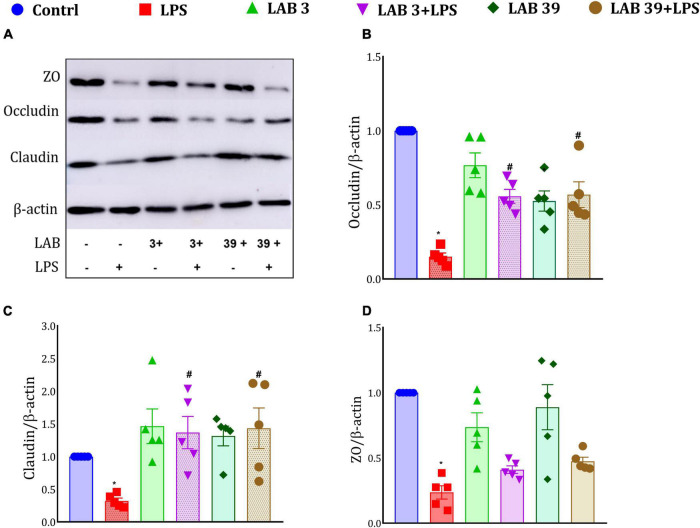
Effect of LAB on the tight junction protein expression: **(A)** Representative Western blotting images; **(B)** expression of occludin relative to β-actin; **(C)** expression of claudin relative to β-actin; and **(D)** expression of zona occludin relative to β-actin. Data were analyzed using one-way ANOVA with Tukey’s *post hoc* test (*p* ≤ 0.05). *Significant relative to control; #significant relative to the LPS group (*N* = 5).

Lipopolysaccharide treatment decreased the protein expression of tight junction proteins (Figure 9A), namely, zona occludin, claudin, and occludin, relative to the control group. Moreover, the LAB-pre-supplemented group LAB3 + LPS and LAB39 + LPS showed higher expression of occludin and claudin proteins than the LPS-treated mice, while no significant difference was observed in the zona occludin expression of these groups. Furthermore, *per se* supplementation of LAB3 and LAB39 also elevated the expression of tight junction proteins relative to the control group (Figures 9B–D).

### Correlation Matrix of Different Parameters Assessed in the Study

**FIGURE 10:**
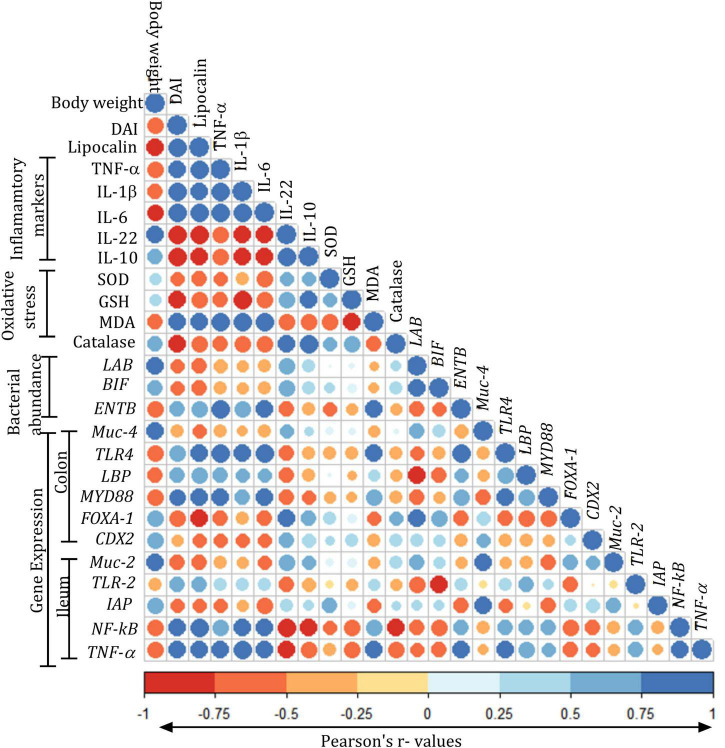
Pearson’s correlation matrix of various parameters assessed in the study: Color gradients were given in terms of “*r*” values from Pearson’s correlation analysis for body weights (g); disease activity index (DAI); lipocalin (ng/ml); tumor necrosis factor-alpha (TNF-α, pg/ml); interleukin (IL)-1 β, IL-6, IL-22, and IL-10 (pg/ml); oxidative stress parameters: superoxide dismutase (SOD, U/mg protein), malondialdehyde (MDA, nM/mg protein), reduced glutathione (GSH, pM/mg protein), superoxide dismutase (SOD, U/mg protein), catalase (U/mg protein); expression of Lactobacillus (LAB), Bifidobacteria (BIF), *Enterobacteriaceae* (ENTB), muc gene (*MUC-4*), toll-like receptor-4 (*TLR-4*), LPS-binding protein (*LBP*), myeloid differentiation factor (*MYD88*), forkhead box protein (*FOXA-1*), caudal type homeobox 2 (*CDX2*) in the colon, expression of genes muc gene (*MUC-2*), toll-like receptor (*TLR2*), intestinal alkaline phosphatase (*IAP*), nuclear factor-κ beta (*NF-kB*), and tumor necrosis factor-alpha (*TNF-*α) in the ileum. All the values are expressed as mean.

Correlation analysis of the various parameters suggested a positive correlation of DAI with lipocalin, *TNF-*α, *IL-1*β, *IL-6*, *TLR4*, and *TLR2* and negative correlation with *Muc-2*, *Muc-4*, *FOXA1*, and *CDX2.* The oxidative stress levels of SOD, GSH, and catalase were negatively correlated with *TNF-*α, *IL-1*β, and *IL-6*. The abundances of *LAB* and *BIF* showed positive correlation with *FOXA-1*, *CDX2*, *Muc-4*, and *Muc-2* and a negative correlation with *NF-kB* and TNF-α. *ENTB* showed positive correlation with *TLR4*, *MYD88*, *LBP*, *NF-kB*, and *TNF-*α. The expression of Muc-2 and Muc-4 showed a positive correlation with *FOXA-1*, *CDX-2*, and *IAP*, while they showed a negative correlation with *TLR4*, *LBP*, *IAP*, *NF-kB*, and *TNF-*α. The expression of *LBP* showed a positive correlation with *TNF-*α, *IL-1*β, and *IL-6* and a negative correlation with *FOXA-1*, *CDX-2*, and *IAP*. The expression of *IAP* showed a positive correlation with *Muc-4*, *Muc-2*, *FOXA-1*, and *CDX-2*, whereas it showed a negative correlation with the lipocalin *TNF-*α, *IL-1*β, *IL-6*, and *TLR4* (Figure 10).

## Discussion

Probiotics confer health benefits to the host, mainly by improving the gut immune system, gut barrier integrity, pathogen exclusion, and secretion of host antimicrobial components, such as β-defensins, and finally establishing the homeostasis in the gut system (Maldonado Galdeano et al., 2019). Many *in vitro* and *in vivo* studies have emphasized the anti-inflammatory potential of lactic acid bacterial strains (Cristofori et al., 2021); however, reports on the anti-inflammatory potential of lactic acid bacterial strains of Indian origin are scarce, despite the rich microbial diversity. Hence, the search for novel probiotic strains with specific health benefits is always considered important. Therefore, in the present study, the potential role of five indigenous lactic acid bacterial strains isolated from different sources in ameliorating LPS-induced inflammation in C57BL/6 mice was evaluated. The clinical condition of inflammation was induced by a single intraperitoneal injection of LPS as 10 mg/kg of body weight on the 14th day in the mice. LPS treatment decreased the body weight and colonic length, followed by its weight, and profoundly increased the disease activity index. The mice pre-supplemented with the LAB strain were protected from these harmful changes. Our results are in accordance with the previous studies where *Lactobacillus reuteri* CRL1101 and *Lactobacillus plantarum* CECT 7315/7316 downregulated the levels of *IL-1*β, *IL-6*, and *TNF-*α and attenuated the LPS-induced inflammation in mice (Vilahur et al., 2015; Gao et al., 2016).

Lipopolysaccharide treatment augmented the production of reactive oxygen species (ROS), which causes an imbalance between the antioxidant defense system and the ROS generation (Savignac et al., 2016; Hu and Li, 2018), which is an important event in inflammation. In this study, the LPS group showed lower SOD, GSH, and catalase and increased levels of MDA, while reduced levels of lipid peroxides and elevated levels of GSH in LAB-pre-supplemented groups suggested protection from the LPS-induced oxidative stress and intestinal mucosal maladies, which is in congruence with earlier reports (Gao et al., 2016; Son et al., 2018). It has been established that LPS dampens the intestinal epithelial permeability and elicits a pro-inflammatory response, resulting in the progression of chronic low-grade inflammation *via* activating macrophages (Cai et al., 2020). In our study, LPS treatment increased the levels of pro-inflammatory markers and fecal lipocalin levels and altered the expression of tight junction proteins and expression of *Muc-4* relative to the LAB-pre-supplemented groups. Interestingly, in the LAB *per se*-supplemented groups, there was no increase in the inflammatory markers but showed enhanced expression of *Muc-4* and tight junction proteins. These findings are in agreement with those of a study that observed the role of *Muc4* in mitigating inflammation-associated tumorigenesis using an *Muc4* novel mouse model (Das et al., 2016). The LPS-induced inflammation is triggered due to its binding to the LPS/LPS-binding protein (LBP) complex, which then binds to TLR-4 receptors. It is then followed by binding with MYD88, which then triggers the nuclear translocation of the phosphorylated NF-kB and then subsequent production of the pro-inflammatory markers (Page et al., 2022). In this study, LPS caused an increased expression of *LBP*, *TLR-4*, *FOXC-2*, *TNF-*α, *NF-kB*, and *Cox2* in the colon and ileum, which is in accordance with earlier reports (Hiraki et al., 2020). We have also observed a decreased expression of intestinal alkaline phosphatase (*IAP*), which encodes for a brush border enzyme required for gut homeostasis. *IAP* detoxifies LPS and other pro-inflammatory nucleotides, regulates bicarbonate secretion, and regulates the gut microbiome and thus helps in maintaining and protecting the gut homeostasis (Singh et al., 2020). Recent studies suggested ileal expression of *IAP* and its phosphatase activity provide protection against metabolic endotoxemia (Fawley and Gourlay, 2016). Interestingly, in this study, the expression of *IAP* in the ileum was found to be high in LAB-pre-supplemented groups relative to the LPS group. Thus, we propose that LAB supplementation might have dephosphorylated the LPS and hence help in reducing the inflammation.

Dysbiosis in microbiota composition in the gut often leads to the aberrant mucosal immunity. In the present study, LPS caused the dysbiosis of the gut bacteria where the abundances of Bacteroidetes, *Lactobacillus*, *Bifidobacteria*, and *Akkermansia* were decreased and the abundances of Gram-negative bacteria *Enterobacter*, *Klebsiella*, *E. coli*, and *Salmonella* were increased, which is in congruence with earlier studies (Fabersani et al., 2017; Ladda et al., 2021). Variations in the gut microbial composition in LPS-treated animals might have contributed to inflammation. Thus, it is associated with a decreased production of short-chain fatty acids (SCFAs), which are important for gut health and improved immune functions. SCFAs, mainly acetate, lactate, propionate, and butyrate, help in differentiation and intestinal epithelial cell proliferation and have different metabolic functions, as many researchers have appreciated their role in inflammation (Wan Saudi and Sjöblom, 2017). In the present study, correlation analysis also suggested a positive association of TNF-α, IL-6, and IL-1β with the lipocalin, *TLR-4* and *TLR-2* and the enhanced abundances of Gram-negative bacteria mainly *ENTB*, while pre-supplementation with LAB showed a negative correlation with *TLR-4*, *TLR-2*, *LBP*, *MYD88*, *NF-kB*, and *TNF-*α, which is also supported by previous findings (Ren et al., 2020).

Taken together, all the five LAB treatment groups conferred protection against LPS-induced gut inflammation and dysbiosis, but LAB3 and LAB39 strains were found to be more potent in preventing LPS-induced inflammatory stress (Anhê et al., 2021), gut bacterial dysbiosis (Lobionda et al., 2019), and promoting overall levels of total SCFA production (LeBlanc et al., 2017), as suggested by the hierarchical cluster analysis (Metsalu and Vilo, 2015) of different treatment groups, which is in congruence with earlier reported studies. Hence, among LAB3 and LAB39, the effect of LAB39 was better as it promoted higher expression of MUC-4 and tight junction protein relative to LAB3. Therefore, LAB3 and LAB39 strain potential could be explored further in mitigating inflammatory diseases and associated conditions.

## Conclusion

This study led to the prioritization of *Lacticaseibacillus rhamnosus* LAB3 and *Lactiplantibacillus plantarum* LAB39 as putative probiotics among the five listed strains as they are more potent in preventing LPS-induced gut inflammation and the selected gut bacterial dysbiosis. The protective effects could be due to the following factors: (i) enhanced tight junction protein levels, (ii) beneficial modulation of selected gut bacteria and suppression of gut pathogens, and (iii) enhanced production of SCFAs, which in turn caused immunomodulatory functions. Taken together the study suggests that indigenous LAB having anti-inflammatory potential could be used as a potential live bacteriotherapy in maintaining gut homeostasis against inflammatory disorders.

## Data Availability Statement

The original contributions presented in this study are included in the article/Supplementary Material, further inquiries can be directed to the corresponding author.

## Ethics Statement

The animal study was reviewed and approved by Institutional Animals Ethics Committee (IAEC), National Agri-Food Biotechnology Institute (NABI), Mohali, Punjab (NABI/2039/CPCSEA/IAEC/2020/11).

## Author Contributions

RB contributed to the conceptualization, methodology, investigation, analysis, and writing the original draft. SS performed the investigation and SCFA analysis. SB contributed to the ethical approval for isolation of bacteria and animal study design. MB contributed to the reviewing, editing, and supervising the manuscript, and resources. KK contributed to the conceptualization, funding acquisition, validation, reviewing and editing the manuscript, supervision, and resources. All authors contributed to the article and approved the submitted version.

## Conflict of Interest

The authors declare that the research was conducted in the absence of any commercial or financial relationships that could be construed as a potential conflict of interest.

## Publisher’s Note

All claims expressed in this article are solely those of the authors and do not necessarily represent those of their affiliated organizations, or those of the publisher, the editors and the reviewers. Any product that may be evaluated in this article, or claim that may be made by its manufacturer, is not guaranteed or endorsed by the publisher.
