# Supplementary material for: Lactic Acid Bacterial Supplementation Ameliorated the Lipopolysaccharide-Induced Gut Inflammation and Dysbiosis in Mice
Source: Front Microbiol. 2022 Jun 13;13:930928. doi: 10.3389/fmicb.2022.930928 (PMC9235405; doi:10.3389/fmicb.2022.930928)
Supplement: Supplementary file 1 [file Data_Sheet_1.docx]

**Supplementary Table S1: List of primers used for qPCR in ileum and colon tissue**

| **S.NO** | **List of genes** | **Forward** | **Reverse** |
| --- | --- | --- | --- |
| 1 | ***CCL5*** | ATCATCCTCACTGCAGCCG | TTCTCTGGGTTGGCACACAC |
| 2 | ***MUC-2*** | GGCCTCACCACCAAGCGTCC | TGGGCTGGCAGGTGGGTTCT |
| 3 | ***MUC-4*** | TTGCACCTGTCCCCCCTGCCT | GTTCGCCACCGAGGCGTTGA |
| 4 | ***TNF-α*** | AGCCTGTAGCCCACGTCGTA | GGCACCACTAGTTGGTTGTCTTTG |
| 5 | ***MYD88*** | TCGACGCCTTCATCTGCTAC | CCATGCGACGACACCTTTTC |
| 6 | ***TLR4*** | GCTTTCACCTCTGCCTTCAC | TCAGCAGGGACTTCTCAACC |
| 7 | ***TLR2*** | CCCTCAGTCTTGGAGTGTCA | CCTGCTCGCTGTAGGAAACA |
| 8 | ***LBP*** | GTTACCGCCTGACTCCAACA | GAGCTGGGCAGGATCACAAA |
| 9 | ***β-actin*** | TGGGCAGGTCATCACGATTG | TACATGGTAGTGCCCCCAGA |
| 10 | ***GAPDH*** | ACCCAGAAGACTGTGGATGG | TTCTAGACGGCAGGTCAGGT |
| 11 | ***Occ*** | GGCAAGCGATCATACCCAGA | TCATAGTGGTCAGGGTCCGT |
| 12 | ***ZO*** | TAACTTGGGGAGGGAGGGTC | GGTAAGGCATTCCTGCTGGT |
| 13 | ***Cldn-1*** | ATGTGGATGGCTGTCATCGG | CAGGAGCAGGAAAGTAG |
| 14 | ***NF-KB*** | GAGGTCTCTGGGGGTACCAT | TTGCGGAAGGATGTCTCCAC |
| 15 | ***DEFB1*** | TCCCAGATGGAGCCAGGT | AGCTGGAGCGGAGACAGA |
| 16 | ***IL-10*** | CCAGAGCCACATGCTCCTA | TTCTGGGCCATGCTTCTC |
| 17 | ***Cox-2*** | CTCAGCCATGCAGCAAATCC | TCCCTTGAAGTGGGTCA |
| 18 | ***CDX2*** | GCAGAGCCAAGGAGAGGAAA | CTTGCAAGGAGGTCACAGGA |
| 19 | ***Galnt1*** | GGGAAACCAGTCGTCATTCC | GTGCTCCATGCCTCATTGTG |
| 20 | ***FOXA1*** | GAGAGAAAAAAUCAACAGC | GCUGUUGAUUUUUUCUCUC |
| 21 | ***FOXC-2*** | CTCCTTTGCGTTTCCAGTGA | ATTGGTCCTTCGTCT TCGCT |
| 22 | ***IL-12*** | GAAGACCTGTTTACCACT | TGATGGCCTGGAACTCTGTC |
| 23 | ***IAP*** | CTCATCTCCAACATGGAC | TGCTTAGCACTTTCACGG |

**Abbreviations**:

**CCL5**= Chemokine ligand 5; **MUC-2**= Mucin-2/4; **IL-10/12**= Interleukin-/10/12; **TNF-α** =Tumor necrosis factor alpha; **MYD88**= Myeloid differentiation primary response 88; **NF-kB**= Nuclear Factor-B; **TLR-4/2**= Toll like receptor; **LBP**= LPS binding protein; **β-actin** = β-actin; **Occ**= Occludin; **ZO**= Zona occludens; **Cldn**= Claudin; **Cox-2**= Cyclyoxygenase-2; **DEFB1**= Defensin beta 1; **FoxA1/C2**= Forkhead box A1/C2; **Cdx2**= Caudal Type Homeobox 2; **Galnt1** = GalNAc transferase; **IAP**= Intestinal Alkaline Phosphatase

**Supplementary Table S2: List of primers used for qPCR in bacterial abundance estimation**

| S.NO | Bacterial primers | Forward 5’-3’ | Reverse 5’- 3’ |
| --- | --- | --- | --- |
| 1 | *AKK* | AACGAACGCTGGCGGCGTGGATAAGACAT | CATCCCAGTTACCAGTCTCACCTTAGGACCCT |
| 2 | *BACT* | ACGCTAGCTACAGGCTTAACA | ACGCTACTTGGCTGGTTCA |
| 3 | *BIF* | GATTCTGGCTCAGGATGA | CTGATAGGACGCGACCCC |
| 4 | *E.COLI* | AAGCTTGCTCTTTGCTGACG | CCGTTACCCCACCTACTAGC |
| 5 | *ENTB* | CATGACGTTACCCGCAGAAG | CTCTACGAGACTCAAGCTTG |
| 6 | *FIRM* | GCGTGAGTGAAGAAGT | CTACGCTCCCTTTACAC |
| 7 | *KLEB* | GCAAGACCAAAGTGGGGGA | CATGGCTGCATCAGGCTTGCGC |
| 8 | *LAB* | CACCGCTACACATGGAG | AGCAGTAGGGAATCTTCCA |
| 9 | *PREV* | GGTGTCGGCTTAAGTGCCAT | CGGAYGTAAGGGCCGTGC |
| 10 | *ROS* | GCGGTRCGGCAAGTCTGA | CCTCCGACACTCTAGTMCGA |
| 11 | *SAL* | ACGTCGCAAGACCAAAGAGG | GGCATGGCTGCATCAGGC |
| 12 | *CLOST* | ACATCCCTCTGACCGGTGTA | CGTGTTATCCACGGCAGTCT |
| 13 | *ENT* | CCCTTATTGTTAGTTGCCATCATT | ACTCGTTGTACTTCCCATTGT |
| 14 | *TB* | GCAGGCCTAACACATGCAAGTC | CTGCTGCCTCCCGTAGGAGT |

***Abbreviations:***

***AKK****= Akkermansia;* ***BACT****= Bacteroidetes;* ***BIF****= Bifidobacterium; FIRM= Firmicutes;* ***LAB****= Lactobacillus;* ***PREV****=Prevotella;* ***E. COLI****= Escherichia coli;* ***ENTB****= Enterobacteriace;* ***ROS****= Roseburia spp;* ***Sal****= Salmonella;* ***CLOST***= Clostridium spp; **KLEB**=Klebsiella; **TB**= Total bacteria; **ENT**=Enterobacter


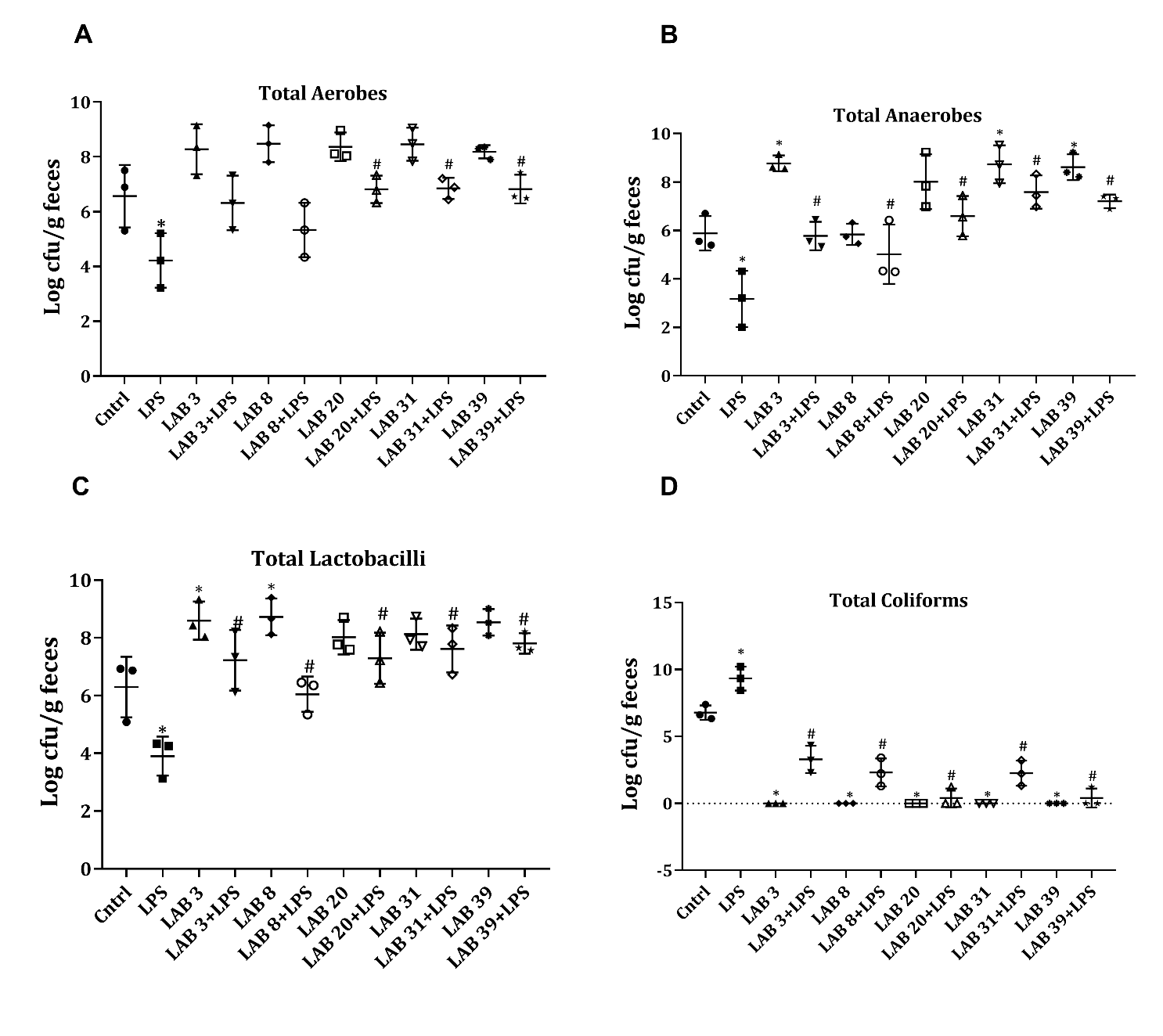


**Figure S1**: **Effect of LAB supplementation on the fecal microbial counts:** Feces from each mouse was collected on 15^th^ day. (A) Total aerobes on nutrient agar; (B) Total anaerobes on anaerobic agar; (C) Total Lactobacilli on MRS agar; (D) Total coliform on violet red agar. Data was analyzed by one-way ANOVA with Tukey’s Post-hoc test (P ≤ 0.05). *significant relative to control; ^#^significant relative to LPS treated animals (N =3).


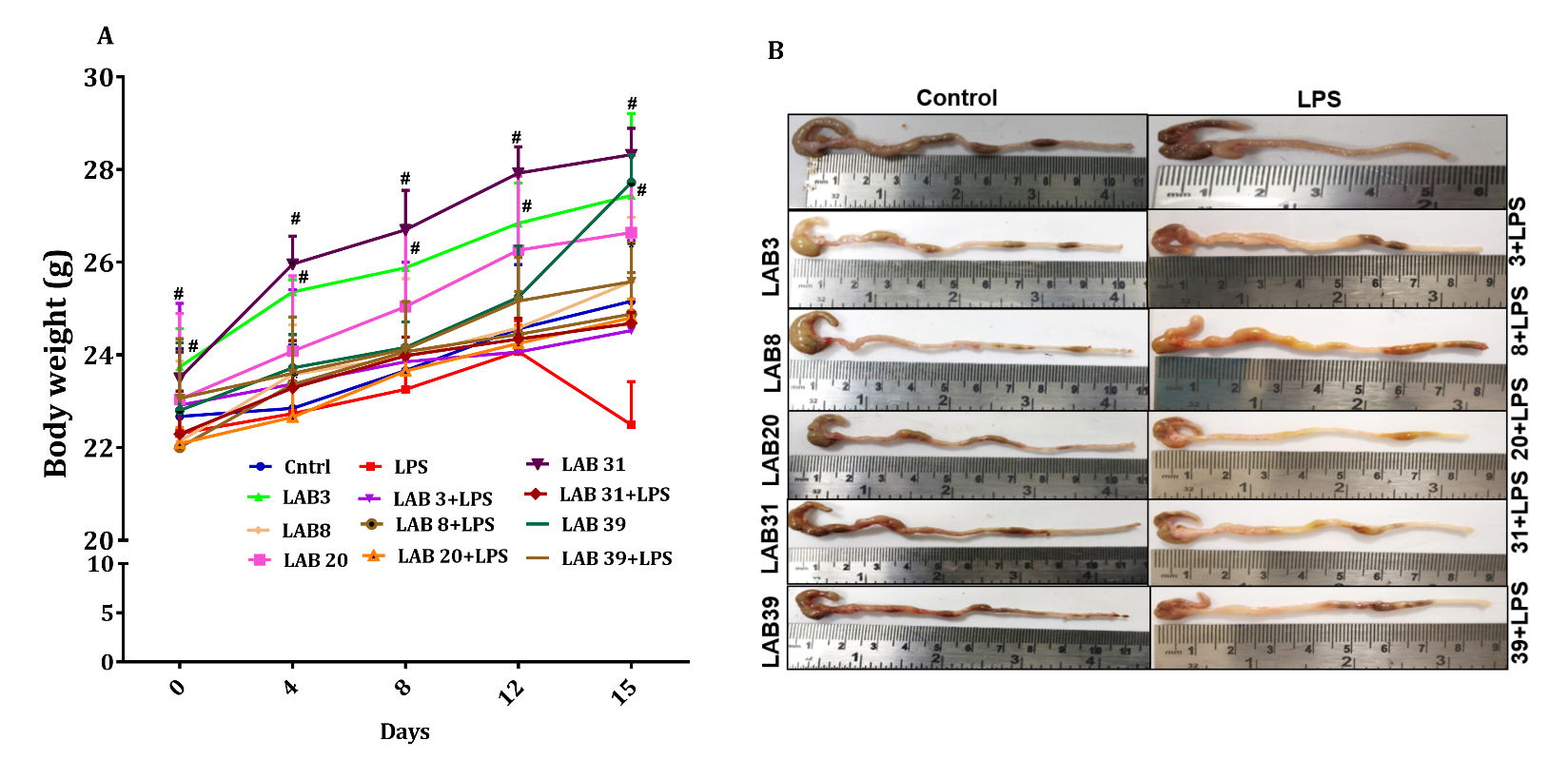


**Figure S2**: **Effect of LAB supplementation on body weight and colon length:** (A) Body weight; (B) Representative images of the colon. Data was analyzed by one-way ANOVA with Tukey’s Post-hoc test (P ≤ 0.05). *significant relative to control; ^#^significant relative to LPS treated animals (N = 5).


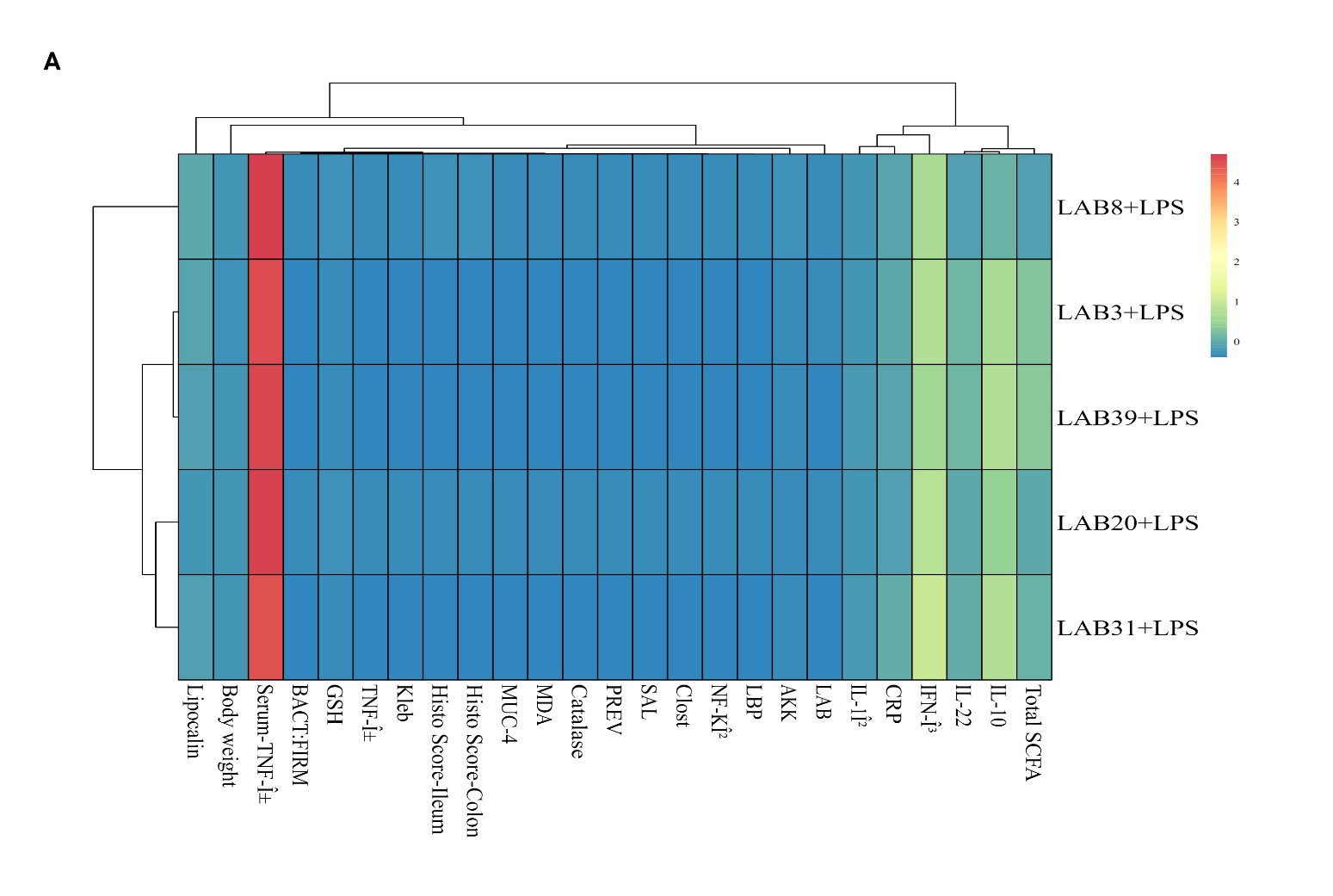


**Figure S3**: **Hierarchical clustering of all five pre LAB-supplemented groups before the LPS treatment.** Heat map was produced for the different parameters used in the study using correlation distance and average linkage. The rows are in the heat map represent different treatment groups and columns represent the different parameters.


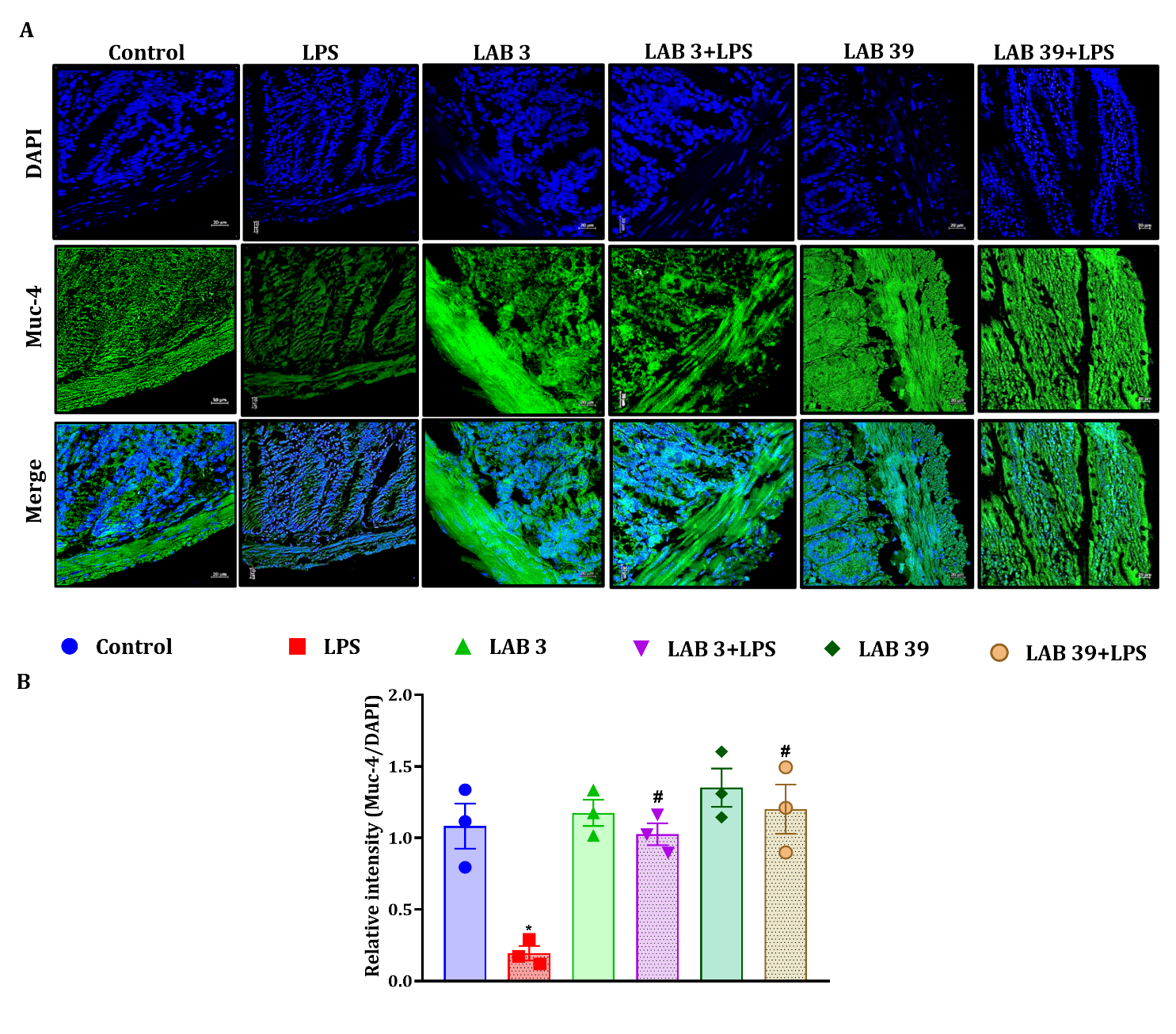


**Figure S4: Effect of LAB supplementation on Muc-4 protein expression:** (A) Immunohistochemistry (fluorescence)– representative images of Muc-4; (B) Fluorescence intensity analysis (antibody signal normalized to DAPI. Data was analyzed using one-way ANOVA with Tukey’s Post-hoc test (p≤0.05). *significant relative to control; # significant relative to LPS group (N=3 for IHC).
